# Effect of rhTSH on Lipids

**DOI:** 10.3390/jcm9020515

**Published:** 2020-02-14

**Authors:** Alessandro P. Delitala, Angelo Scuteri, Margherita Maioli, Gavino Casu, Pierluigi Merella, Giuseppe Fanciulli

**Affiliations:** 1 U.O.C. Medicina Interna 2, Azienda Ospedaliero-Universitaria di Sassari, 07100 Sassari, Italy; 2 Department of Surgical, Medical and Experimental Sciences, University of Sassari, 07100 Sassari, Italy; d341elefante@virgilio.it (A.S.); gfanciu@uniss.it (G.F.); 3 Department of Biomedical Sciences, University of Sassari, 07100 Sassari, Italy; mmaioli@uniss.it; 4 Center for Developmental Biology and Reprogramming (CEDEBIOR), Department of Biomedical Sciences, University of Sassari, 07100 Sassari, Italy; 5 Istituto di Ricerca Genetica e Biomedica, Consiglio Nazionale delle Ricerche (CNR), Monserrato, 09042 Cagliari, Italy; 6 Cardiology Unit, Ospedale San Francesco, 08100 Nuoro, Italy; gcasu61@gmail.com (G.C.); pierluigi9@hotmail.it (P.M.)

**Keywords:** rhTSH, lipids, TSH, total cholesterol, triglycerides, nonHDL cholesterol

## Abstract

Background: Subclinical hypothyroidism is associated with increased blood lipid levels. However, the exact role of thyrotropin (TSH) alone is not clear. In order to clarify this point, we analysed the acute effect of recombinant human TSH (rhTSH) administration on lipid levels. Methods: Sera of 27 premenopausal women with well-differentiated thyroid cancer were analysed. Patients that underwent a total thyroidectomy, ablation with ^131^I (Iodine 131) and rhTSH administration as a part of routine follow-up American Thyroid Association guidelines were included. The protocol consists of 2 intramuscular injections of 0.9 mg of rhTSH, performed on day 1 day and day 2, with blood collection on day 1 (before rhTSH administration), and day 5. TSH, free thyroxine, total cholesterol, low-density lipoprotein cholesterol (LDLc), high density lipoprotein cholesterol (HDLc), and triglycerides were assessed in all the samples, before and four days after the first administration of rhTSH. Results: Total cholesterol and triglycerides significantly increased after stimulation of rhTSH (respectively, 192 ± 33 vs. 207 ± 26, *p* = 0.036 and 72 ± 23 vs. 85 ± 23, *p* = 0.016). LDLc and HDLc showed comparable concentrations before and after the test (respectively, 115 ± 27 vs. 126 ± 22, *p* = 0.066, and 62 ± 15 vs. 64 ± 15, *p* = 0.339), while non-HDLc increased after stimulation (130 ± 30 vs. 143 ± 25, *p* = 0.045). Conclusion: TSH has a direct effect on total cholesterol, triglycerides, and nonHDLc. Explanation of these phenomena will require additional studies.

## 1. Introduction

Thyroid hormones regulate different processes in the human body and the effects of their action are more evident during dysfunction of the thyroid gland, affecting processes from the cardiovascular system to cognition level [1,2,3]. Disorders of the thyroid gland are frequently found in the general population [4], with chronic autoimmune disease the most common disorder, which can have a role in the papillary thyroid cancer [5]. Subclinical hypothyroidism, defined as increased thyrotropin (TSH) level and free hormones within the normal range, is associated with atherosclerosis and worse lipid metabolism [6]. On the other hand, subclinical hyperthyroidism, diagnosed when TSH is below the reference range and free thyroxine (FT4) within the normal range, may predispose to cardiac arrhythmias, mainly atrial fibrillation and increased arterial stiffness [7,8].

While the serious effects of overt hypothyroidism (increased TSH and low free hormones) are well acknowledged, different studies were focused on the association between subclinical hypothyroidism and increased serum lipid concentration. Some of them found an inverse association with FT4, while others a positive and direct effect of TSH (e.g., increased lipids) [9]. The issue is further puzzled by the heterogeneous results found for different classes of lipids. Indeed, some studies found and association between thyroid hormone and total cholesterol [10], while others reported with total cholesterol, low density lipoprotein cholesterol (LDLc), triglycerides [11,12], and even with high density lipoprotein cholesterol (HDLc) [13]. Additional studies also tested the effect of levothyroxine (LT4) therapy on lipids, with different results [14,15]. However, the question of whether the lipid-lowering effect is mediated by FT4 alone or in combination with TSH is still unanswered.

The hypothesis that TSH can directly increase the lipids level is relatively recent, and based on associated studies not only in patients with increased TSH level (e.g., subclinical/overt hypothyroidism), but also in subjects with TSH within the normal reference range [16]. Indeed, TSH can contribute to increase LDLc by increasing the Proprotein convertase subtilisin/kexin type 9 (PCSK9) [17].

Patients affected by differentiated thyroid cancer give the unique opportunity to test whether different concentrations of TSH can increase lipids concentration. Indeed, these subjects, after surgery and ^131^I therapy, have constant FT4 level due to their LT4 supply, which is equal every day. Recent evidence also suggests an association between TSH and hypercholesterolemia in female patients with differentiated thyroid cancer receiving LT4 after total thyroidectomy [18]. As a follow up of the disease, they often perform a stimulation test with recombinant human TSH (rhTSH), which has been used over the last two decades as a safe alternative to the thyroid hormone withdrawn. Injection of rhTSH allows having increased TSH concentration over few days with stable FT4 level.

In this study, we aimed to investigate the role of rhTSH on lipids by using a sample of pre-menopausal women with well-differentiated thyroid cancer, treated with total thyroidectomy and ^131^I remnant ablation.

## 2. Experimental Section

### 2.1. Material and Methods

Sera of premenopausal women with differentiated thyroid who underwent rhTSH protocol were used. This standardised protocol is performed as a part of routine follow-up, according American Thyroid Association guidelines for thyroid cancer [19], 12 months (12 ± 2 months in our Centre), after ablation with ^131^I (following total thyroidectomy). The protocol in collection of fasting blood drawn on day 1 and 5, and intramuscularly injection of 0.9 mg of rhTSH performed on two consecutive days (day 1 and day 2). The rhTSH protocol does not require LT4 withdrawal.

Blood samples were assayed for TSH, FT4, thyroglobulin, antibody against thyroglobulin, total cholesterol, LDLc, HDLc, and triglycerides, using sera from day 1 and day 5.

Serum TSH, FT4 were measured using the Immulite 2000 platform. Reference values were, respectively 0.35–4.94 uUI/mL and 0.7–1.48 ng/dL.

Triglycerides and total cholesterol were determined by an enzymatic method (Abbott Laboratories ABA-200 ATC Biochromatic Analyzer, Irving, TX, USA). High density lipoprotein cholesterol (HDL) was determined by dextran sulphate–magnesium precipitation. LDLc was calculated by the Friedewald formula: LDLc = [(Total Cholesterol − HDLc) − (triglycerides/5)].

Given the origin of the sera employed in the study (residual samples temporarily stored in the local blood bank -Internal Medicine Department, Sassari, Italy-, and otherwise destined for disposal), identified only by a letter (A, female) and a number (age), resulted an absolute inability to identify the subject in order to obtain the consent.

### 2.2. Statistical Analysis

A paired *t*-test was used to assess the differences in each variable before and after rhTSH stimulation. Statistical significance was set at *p* values less than 0.05 in Stata 12.

## 3. Results

Sera of 27 females (mean age 39.4 ± 8 years) were employed in this study. Descriptive characteristics of the results, before and after rhTSH, are described in Table 1. Mean age of the sample was 39.4 ± 8.0 years. Plasma TSH increased after injection of TSH (0.43 ± 0.23 vs. 12.43 ± 4.53, *p* < 0.001), while FT4 values were comparable (1.10 ± 0.21 vs. 1.10 ± 0.22, ns). Total cholesterol and triglycerides significantly increased after stimulation of rhTSH (respectively, 192 ± 3 vs. 207 ± 26, *p* = 0.036 and 72 ± 23 vs. 85 ± 23, *p* = 0.016). LDLc showed an increase of borderline statistical significance (115 ± 27 vs. 126 ± 22, *p* = 0.066), while HDLc showed comparable concentration before and after the test (62 ± 15 vs. 64 ± 15, *p* = 0.339), while non-HDLc increased after stimulation (130 ± 30 vs. 143 ± 25, *p* = 0.045).

To test lipids difference across age, we split the sample accordingly to mean age, as reported in Table 2. Women older than 39.4 years before administration of rhTSH had increased level of total cholesterol and LDLc compared to younger women (*p* < 0.001 for both). After rhTSH, women aged 39.4 or older still have increased total cholesterol and LDLc (*p* < 0.01 for both). HDL and triglycerides were comparable indecently from mean age groups, before and after rhTSH.

## 4. Discussion

In this study, we found that administration of rhTSH acutely increases lipids levels. In particular, we observed an increase of total cholesterol, triglycerides, and non-HDLc, while LDLc and HDLc showed no significant variations.

The effect of thyroid hormone on lipid metabolism is complex and poorly understood. Lipid metabolism is regulated by different enzymes, which are regulated by different specific gene and hormones [20,21] and lack of thyroid hormone causes elevation in total cholesterol and LDLc by changes in the synthesis, metabolism, and mobilisation of lipids [22].

Thyroid hormone induces the transcription of LDL receptor gene in the liver and the expression of hydroxymethylglutaryl coenzyme A reductase (HMGCR), therefore increasing cholesterol synthesis. This means that in case of hypothyroidism, the hepatic cholesterol synthesis is reduced. At the same time, triiodothyronine increases the expression of the sterol regulatory element-binding protein-2 (SREBP-2), which increases the expression of LDL receptor. Lack of thyroid hormone cause a reduction of SREBP-2 and, consequently, a reduction of LDL receptor, causing hypercholesterolemia. Hypothyroidism causes also a reduction of cholesterol ester transfer protein (CETP), which may be related to alteration in HDLc levels [23]. The importance of these reports is due to the high prevalence of disorders of thyroid gland [4], which can have a deep impact on the cardiovascular risk.

The lipid-lowering effect of thyroid hormone has been documented also by its use as therapeutic agent, the so-called thyromimetics. Eprotirome, one of the most studied thyromimetic drug, stimulates triiodothyronine (T3) receptor-β isoform, and results in a decreases triglycerides, LDLc, apoB, and lipoprotein lipase a [24].

Recent evidence suggests that TSH was associated with lipids, independently of FT4. Gong et al. demonstrated that patients with subclinical hypothyroidism have increased serum PCSK9 levels, whose expression is enhanced in rhTSH-treated HepG2 cells through the activation of SREBP1c and SREBP2. Consequently, LDL uptake is decreased resulting in increased plasma concentration of LDL [17]. Additional studies suggest that TSH limits hepatic bile acids synthesis through SREBP2 signalling pathway, thus reinforcing the hypothesis that TSH may regulate lipids metabolism independently by thyroid hormone [25].

A recent study reported the effect of rhTSH on 82 patients with differentiated thyroid cancer, previously treated with total thyroidectomy and ^131^I remnant ablation [26]. The analyses showed a decrease of HDL, from 0.92 to 0.81 mmol/L (*p* < 0.001), and increase of triglycerides from 1.98 to 2.50 mmol/L (*p* < 0.001), both associated with the decrease of T3 level. In this study, authors included male and female and the median age was 44. Thus, the difference result obtained in our analyses can be justified by the gender and age difference. Indeed, in a previous study, Delitala et al. showed that the relation between TSH and lipid parameters was gender-specific, and further demonstrated that the lack of estrogen could contribute to this association [27]. The association between thyroid hormone, as well as many cardiovascular diseases [28], could have a gender difference [29]. Anyway, we are not able to confirm that the lowering effect of rhTSH was mediated by T3, as reported by the previous study, because we did not assess it. We can speculate a direct effect of TSH on lipids, and not mediated by cytokine. Indeed, a previous report found that adipocytokine could interact with thyroid hormone [30,31], but acute effect of rhTSH is ineffective in changing leptin levels [32].

We did not found any significant change in LDLc and HDLc. LDLc has a half-life of 3.5 days [33] and the change in activity of hydroxymethylglutaryl coenzyme A (HMG-CoA) and hepatic microsomal cholesterol 7-α-hydroxylase cause a significant reduction in LDL within 1 day [34]. Thus, the lack of association could be due to the size of our sample, not enough ample to detect significant associations, as demonstrated by the borderline statistical significance found in our analysis (*p* = 0.066).

We acknowledged some limitations of the study. First, we cannot ascertain whether blood sample have been collected in a specific phase of menstrual cycle, thus discerning possible effect of higher/different estrogen concentrations. In addition, we did not assess blood sample after the 5th day from the injection of rhTSH, and we can only hypothesise a reduction of lipid concentrations due to the progressive reduction of TSH. However, our study included patients without any residual of the thyroid gland due to surgery and ^131^I therapy, as demonstrated by the absence of circulating thyroglobulin. This provides an ideal model in which FT4 values remain stable during the test, due to constant LT4 supply, then analysing the direct effect of TSH alone on serum lipids. The questions whether different estrogen concentrations (or lack of them, as occurs in menopause) might play ad additive role cannot be answered by the present study.

## 5. Conclusions

We demonstrated that rhTSH can increase total cholesterol, triglycerides, and nonHDLc, independently from FT4 level. The understanding of the exact mechanism(s) through which TSH can stimulate hepatic cell to produce lipids will require additional studies.

## Figures and Tables

**Table 1:** Thyroid hormones and lipid before and after recombinant human thyrotropin stimulating hormone (rhTSH) stimulation.

Variable	Before rhTSH	After rhTSH	*p* Value
TSH (mUI/L)	0.43 ± 0.23	12.34 ± 4.53	<0.001
FT4 (ng/dL)	1.10 ± 0.21	1.10 ± 0.212	ns
Total cholesterol (mg/dL)	192 ± 33	207 ± 26	0.036
LDLc (mg/dL)	116 ± 27	126 ± 22	0.066
HDLc (mg/dL)	62 ± 15	63 ± 15	ns
Triglycerides (mg/dL)	72 ± 23	85 ± 23	0.016
Non-HDLc (mg/dL)	130 ± 30	143 ± 25	0.037

*n* = 27; age 39.4 ± 8 years; rhTSH, recombinant human thyrotropin; TSH, thyrotropin; FT4, free thyroxine; LDLc, low density lipoprotein cholesterol; HDLc, high density lipoprotein cholesterol.

**Table 2:** Lipids difference before and after administration of rhTSH stratified by mean age.

Variable	Pre-rhTSH		Post-rhTSH	
	Age ≤ 39.4	Age > 39.4	Age ≤ 39.4	Age > 39.4
Total cholesterol (mg/dL)	173	210 *	191	219 ^#^
LDLc (mg/dL)	100	131 *	114	135 ^#^
HDL (mg/dL)	59	64	61	67
Triglycerides (mg/dL)	69	73	83	86

LDLc, low density lipoprotein cholesterol; HDLc, high density lipoprotein cholesterol. * Age ≤ 39.4 vs. age > 39.4 before administration of rhTSH, *p* < 0.001 ^#^ Age ≤ 39.4 vs. age > 39.4 after administration of rhTSH, *p* < 0.01.
